# Risk factor analysis for the development and progression of retinopathy of prematurity

**DOI:** 10.1371/journal.pone.0219934

**Published:** 2019-07-18

**Authors:** Ji Woong Chang

**Affiliations:** 1 Department of Ophthalmology, Inje University Ilsan Paik Hospital, Inje University College of Medicine, Goyang, Korea; 2 Graduate Program in Cognitive Science, Yonsei University, Seoul, Korea; Children's Hospital Boston, UNITED STATES

## Abstract

**Purpose:**

To classify the risk factors that contribute to the development versus progression of retinopathy of prematurity (ROP).

**Methods:**

The medical records of premature infants born with a birth weight (BW) less than 1501 g or a gestational age (GA) of 32 weeks or less were retrospectively reviewed. Twenty potential risk factors that may influence the development or progression of ROP were analyzed by univariate and multivariate logistic regression analyses. The progression of ROP was defined as type 1 ROP, threshold ROP, or aggressive posterior ROP for which treatment was recommended.

**Results:**

A total of 324 eyes were included; 157 eyes (48.5%) showed ROP development, and 48 eyes exhibited ROP progression (14.8% of all eyes and 30.6% of the ROP-developed eyes). According to the univariate and multivariate logistic regression analyses, prenatal steroid use, GA, the duration of mechanical ventilation, and respiratory distress syndrome were associated with the development of ROP. However, GA, bronchopulmonary dysplasia, the number of red blood cell units transfused, intraventricular hemorrhage, and periventricular leukomalacia were significantly correlated with ROP progression.

**Conclusion:**

The risk factors that influenced ROP development versus ROP progression were not identical. Evaluating these risk factors during screening of high-risk premature infants will help determine the appropriate timing of examinations and treatment.

## Introduction

Retinopathy of prematurity (ROP) is a vasoproliferative disease of the developing retina of premature infants.[1] Because ROP is the major cause of blindness in preterm infants, screening is scheduled according to gestational age (GA), birth weight (BW), and ROP stage[2] to detect ROP development and progression and to determine the appropriate timing of treatment to prevent blindness.[3] As the visual outcomes of high-risk preterm infants are a serious concern for parents and ophthalmologists, and although the course of ROP and the treatments to reduce vision loss are well known, detecting ROP and determining the optimal treatment plan for high-risk preterm infants is crucial.[4, 5] The incidence of ROP development is approximately 60%, and the rate of ROP progression to a severe status is approximately 15%.[6, 7] However, these rates vary depending on the birth and survival rates of premature infants in each country and on weight and GA at birth. Therefore, examinations and treatment plans for ROP should be carefully devised by considering the risk factors for ROP development and progression, and the risk factors of ROP development must be distinguished from those of ROP progression. ROP is a multifactorial disease, and several risk factors have been analyzed in numerous studies. However, few studies have distinguished the risk factors of ROP development from those of ROP progression. In this study, the potential risk factors for ROP were analyzed to determine which risk factors influence ROP development versus ROP progression.

## Materials and methods

This study was approved by the Inje University Ilsan Paik Hospital Institutional Review Board and was conducted in accordance with the ethical principles for medical research outlined in the Declaration of Helsinki. All medical records were completely anonymized, de-identified and aggregated before data were collected and analyzed. Premature infants born with a BW less than 1501 g or at a GA of 32 weeks or less between 2010 and 2015 were included,[2] and the detailed medical records of these patients were retrospectively reviewed.

Infants with severe systemic diseases or congenital anomalies that could affect ROP development or progression and those with other retinal abnormalities were excluded. The stage of ROP was classified according to the International Classification or Retinopathy of Prematurity.[8] ROP development was defined as the occurrence of any stage of ROP, and ROP progression was defined as ROP that did not resolve spontaneously after ROP development and instead worsened to severe ROP, such as type 1 ROP, threshold ROP, or aggressive posterior ROP; treatment was recommended accordingly.

Twenty potential risk factors that may influence the development and progression of ROP were divided into maternal factors and infantile factors. The infantile factors were further subdivided into birth factors, systemic factors, and brain factors.

### Statistical methods

To identify risk factors for the development versus progression of ROP, each candidate risk factor for ROP development and progression was initially analyzed by univariate logistic regression analysis. Multivariate logistic regression analysis was then performed by stepwise backward removal of risk factors according to the lowest likelihood ratio. The odds ratio and 95% confidence interval associated with each predictor were calculated from the logistic regression models. A P value < 0.05 was considered statistically significant. All analyses were performed using SPSS software (version 18.0, SPSS Inc., Chicago, IL).

## Results

A total of 324 eyes of 162 premature infants who met the inclusion criteria were analyzed in this study. The mean GA at birth was 28.5 weeks, and the mean BW was 1224.9 g. The detailed demographic data are presented in Table 1.

**Table 1:** Baseline characteristics of the participants.

	Mean	Range
Birth weight (gram)	1224.9 ± 376.2	530–2170
Gestational age (weeks)	28.5 ± 3.0	21–36
Male / %	88 / 54.3	

**Fig 1:**
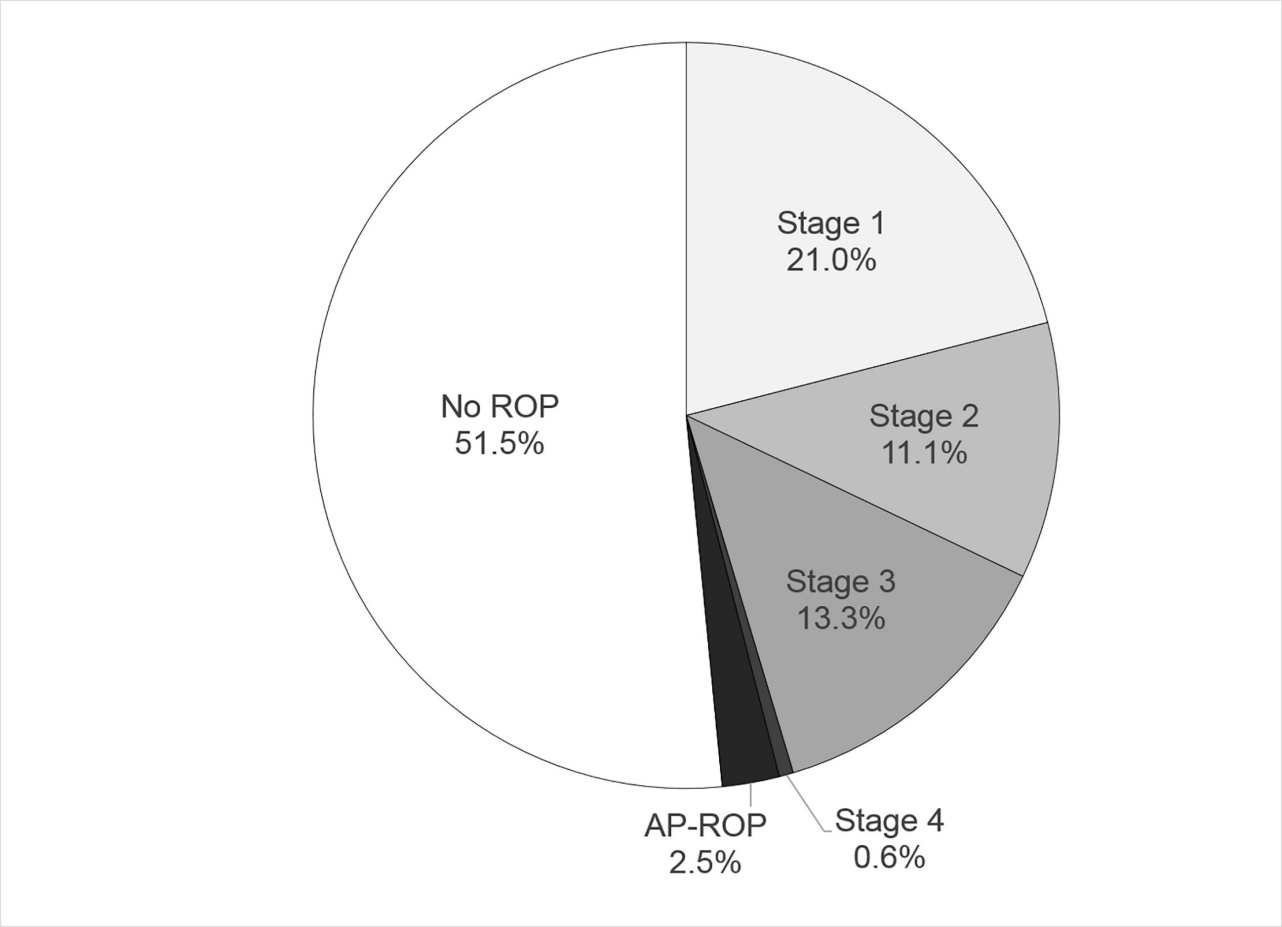
The final stages of retinopthy of prematurity development.

Among the 324 eyes, 157 eyes (48.5%) showed ROP development. A summary of the final stages of ROP is shown in Fig 1. Among the 157 eyes that developed ROP, 48 eyes progressed to severe ROP (14.8% of all eyes and 30.6% of ROP developed eyes). The detailed data of ROP progression are shown in Table 2. The maximum stages of the eyes that showed ROP development but not progression to severe ROP included stage 1 in 68 eyes (21.0%), stage 2 in 31 eyes (9.6%), and stage 3 in 10 eyes (3.1%).

**Table 2:** Progression of retinopathy of prematurity.

	Eyes	% of ROP development (n = 157)	% of total eyes (n = 324)
Type 1 ROP	27	17.2	8.3
Threshold ROP	13	8.2	4.0
AP-ROP	8	5.1	2.5

AP-ROP, aggressive posterior retinopathy of prematurity; ROP, retinopathy of prematurity.

After univariate logistic regression, various factors were identified as significant risk factors for ROP development and ROP progression (Table 3). Multiple logistic regression with the risk factors for ROP development showed that prenatal steroid therapy, GA, the duration of mechanical ventilation, and respiratory distress syndrome (RDS) were significant risk factors. However, multiple logistic regression with the risk factors for ROP progression revealed that GA, bronchopulmonary dysplasia (BPD), the number of red blood cell (RBC) units transfused, intraventricular hemorrhage (IVH), and periventricular leukomalacia (PVL) were significant risk factors (Table 4).

**Table 3:** Univariate logistic regression for the development versus progression of retinopathy of prematurity.

	ROP development				ROP progression			
	P Value^a^	OR	95% CI for the OR		P Value^a^	OR	95% CI for the OR	
*Maternal factors*								
Age at childbirth	0.004^a^	1.078	1.025	1.135	0.187	1.049	0.977	1.127
Diabetes mellitus	0.542	1.381	0.489	3.904	0.887	0.896	0.196	4.103
Hypertension	0.293	0.700	0.360	1.362	0.071	0.261	0.061	1.119
Prenatal steroid therapy	0.002^a^	2.320	1.369	3.931	0.924	0.965	0.466	1.999
*Birth factors*								
Gestational age	<0.001^a^	0.556	0.492	0.629	<0.001^a^	0.601	0.516	0.699
Birth weight	<0.001^a^	0.995	0.995	0.996	<0.001^a^	0.996	0.995	0.998
Multiple births	0.487	1.185	0.735	1.911	0.104	1.693	0.897	3.197
AS at 1 minute	<0.001^a^	0.667	0.588	0.757	<0.001^a^	0.681	0.582	0.796
AS at 5 minutes	<0.001^a^	0.534	0.425	0.670	<0.001^a^	0.579	0.451	0.742
Total mechanical ventilation duration	<0.001^a^	1.042	1.033	1.052	<0.001^a^	1.033	1.023	1.043
*Systemic factors*								
RDS	0.017^a^	12.034	1.565	92.532	0.261	2.932	0.450	19.105
BPD	<0.001^a^	5.009	2.988	8.397	0.022^a^	2.369	1.133	4.954
PDA	0.001^a^	2.286	1.420	3.682	0.018^a^	2.437	1.163	5.105
Chorioamnionitis	0.781	0.919	0.506	1.669	0.764	0.876	0.370	2.077
Sepsis	<0.001^a^	4.300	2.457	7.526	0.003^a^	4.979	1.734	14.296
DIC	0.118	3.629	0.721	18.258	0.086	3.613	0.834	15.648
The number of RBC units transfused	<0.001^a^	1.487	1.353	1.634	<0.001^a^	1.274	1.177	1.378
*Brain factors*								
IVH	0.002^a^	4.367	1.705	11.187	<0.001^a^	6.238	2.677	14.538
Hydrocephalus	<0.001^a^	4.816	2.414	9.609	0.002^a^	3.030	1.499	6.125
PVL	0.364	1.335	0.715	2.494	<0.001^a^	4.100	2.016	8.338

AS = apgar score; BPD = bronchopulmonary dysplasia; CI = confidence interval; DIC = disseminated intravascular coagulation; IVH = intraventricular hemorrhage; OR = odds ratio; PVL = periventricular leukomalacia PDA = patent ductus arteriosus; RBC = red blood cell; RDS = respiratory distress syndrome; ROP = retinopathy of prematurity

^a^P < 0.05.

**Table 4:** Significant risk factors after multivariate logistic regression for the development versus progression of retinopathy of prematurity.

	Development of ROP			
	P Value	OR	95% CI for the OR	
Prenatal steroid therapy	<0.001	4.997	2.237	11.164
Gestational age	<0.001	0.532	0.408	0.693
Total mechanical ventilation duration	<0.001	1.035	1.015	1.055
RDS	0.002	158.748	6.590	3824.021
	Progression of ROP			
	P Value	OR	95% CI for the OR	
Gestational age	<0.001	0.635	0.512	0.787
BPD	0.012	0.253	0.087	0.738
The number of RBC units transfused	0.041	1.112	1.004	1.231
IVH	0.002	3.944	1.655	9.399
PVL	0.005	3.633	1.484	8.892

BPD = bronchopulmonary dysplasia; CI = confidence interval; IVH = intraventricular hemorrhage; OR = odds ratio; PVL = periventricular leukomalacia; RBC = red blood cell; RDS = respiratory distress syndrome; ROP = retinopathy of prematurity.

## Discussion

ROP developed in 48.5% of all eyes, and ROP progressed to a severe status in 14.8% of all eyes and in 30.6% of the ROP developed eyes. The risk factors that influenced ROP development versus ROP progression were not identical.

Prenatal steroids have been increasingly used to prevent RDS and death in preterm neonates.[9] Prenatal steroids are considered to facilitate fetal lung maturation and result in a decreased need for mechanical ventilation and supplemental oxygen.[10] However, the relation between prenatal steroid use and ROP is controversial. Previous studies have demonstrated that prenatal steroid use decreased the incidence of severe ROP[11] or had no effect on the development of severe ROP.[12] In this study, prenatal steroid use was correlated with a higher rate of ROP development but was not associated with ROP progression. Prenatal steroid use is correlated with an increased rate of ROP development likely because prenatal steroids are used when the mother and neonate are in poor condition. The additional statistical analysis showed that the prenatal steroid use group had a lower GA (P < 0.001, t-test) and a longer duration of mechanical ventilation (P = 0.007, t-test). Therefore, prenatal steroid use may not directly induce ROP development but may be indirectly associated with ROP development.

Supplemental oxygen for very preterm infants is critical for their survival but can cause various oxygen toxicities in multiple organs, including the lungs and the brain.[13, 14] Unrestricted use of supplemental oxygen was discovered to be a major cause of ROP.[15, 16] However, even if oxygen saturation is carefully monitored, this parameter is still considered a major risk factor for ROP.[17–19] Various methods can be used to deliver oxygen to preterm infants. In this study, according to the univariate logistic regression, the duration of each method of oxygen delivery was significantly associated with ROP development, and all methods except for nasal cannula application were associated with ROP progression. However, the multivariate logistic analysis revealed that only the duration of mechanical ventilation was an independent risk factor for ROP development, while none of the other oxygen delivery methods were associated with ROP progression. Therefore, the duration of mechanical ventilation is a greater predictive factor for ROP development than the total duration of O2 supplementation or any other method of O2 delivery but is not related to ROP progression.

RDS is a breathing disorder in premature infants and is caused by a surfactant deficiency in the lungs, while BPD is a chronic scarring lung disease that requires prolonged oxygen supplementation. If RDS is prolonged, BPD can develop. BPD develops as a result of lung injury due to oxygen toxicity and mechanical ventilation. Akkoyun et al.[20] reported that RDS was a significant risk factor for the development of stage 1 and 2 ROP. Holmström et al.[18] argued that RDS and BPD were associated with ROP, and stepwise logistic regression revealed that BPD was significantly associated with ROP. In this study, RDS had a significant association with the development of ROP, and BPD was related to the progression of ROP. These results are highly convincing because RDS necessitates supplemental oxygen and can induce hyperoxemia in the developing retina, thus arresting retinal vascular growth. Consequently, the resulting hypoxia of the retina can cause ROP. However, initial supplemental oxygen is not associated with the progression of ROP. In contrast, the chronic lung abnormality BPD requires prolonged supplemental oxygen, which may cause the progression of ROP to a severe status.

Previous studies have shown that blood transfusions influenced ROP development.[21] However, Slidsborg et al.[22] identified blood transfusion as an independent risk factor for ROP requiring treatment, similar to ROP progression in this study. Transfused blood increases adult hemoglobin levels and the iron load in preterm infants. Because adult hemoglobin has a lower affinity for oxygen than fetal hemoglobin, increased oxygen transport by adult hemoglobin increases oxygen delivery to the developing retina, which can reduce angiogenesis. Furthermore, elevated iron loads can induce free radicals. Both situations can induce ROP development or progression.[23] In this study, blood transfusions were not associated with ROP development but affected the progression of ROP.

Abnormalities of central nervous system including IVH, hydrocephalus, and PVL are significantly associated with the progression of ROP. IVH occurs in approximately 20% to 40% of premature infants with a BW less than 1500 g.[24] Holomström et al.[17] and Shah et al.[25] showed an association between IVH and ROP development by univariate analysis but no significant association in a stepwise multivariate analysis. However, Borwn et al.[26] demonstrated that IVH is related to stage 3–4 ROP, which is similar to the result of this study showing that IVH was a significant risk factor for ROP progression. IVH and ROP share similar characteristics: IVH develops due to fragility of the germinal matrix vasculature and fluctuations in cerebral blood flow[27], which is similar to ROP as both pathologies are associated with immature vasculature and an unstable O2 supply.[28] Hydrocephalus in preterm infants primarily results from IVH[29] and may therefore also be related to ROP progression.

PVL refers to hypoxic ischemic damage to the periventricular white matter. Decreased systemic blood pressure and oxygen imbalance cause apoptosis of developing brain cells and induce insufficient myelination of nerve fibers.[30, 31] The pathophysiology of PVL also shares similar characteristics to that of ROP as both are associated with changes in the oxygen balance.[32] ROP progression indicates a continuing problem in retinal vascular growth mainly due to oxygen imbalance, which is also the primary cause of PVL development. Therefore, one can reasonably assume that PVL and ROP progression share a similar risk environment rather than considering that one is a direct risk factor for the other.

Among the various potential risk factors, only GA contributes to both the development and progression of ROP. A low GA has been considered to be the most important risk factor for ROP.[33, 34] GA is a definite indicator of immaturity of the nervous and vascular systems of preterm infants. A lower GA increases the possibility that an infant does not receive critical factors provided by the intrauterine environment, and consequently, the infant is exposed to an unfavorable setting, thus contributing to the risk of ROP.[7].

This study has some limitations. First, all the identified risk factors could not be included in this study. ROP is a multifactorial disease with numerous risk factors. Therefore, a selection bias was possible during the selection of potential risk factors. Second, this study was conducted in one neonatal intensive care unit (NICU). Although the NICU is a regional referral center and all admitted preterm infants receive standardized and advanced treatment, the number of included preterm infants was relatively small. Nevertheless, this study provides a very consistent conclusion and clearly distinguished between the risk factors for the development and progression of ROP.

## Conclusions

The reasons why some factors are related only to the development of ROP while other factors are related only to the progression of ROP remain unclear. Prenatal steroid use, the duration of mechanical ventilation, and RDS, which influence ROP development, are factors that are introduced earlier in the development of preterm infants, whereas BPD, RBC transfusion, and brain abnormalities are later problems in preterm infants who continue to exhibit worsening circumstances. In addition, GA may contribute to the risk of ROP in both early- and later-stage environments. These findings may provide pediatricians and ophthalmologists with valuable information for the screening of high-risk preterm infants, enabling them to determine the risk of ROP development or progression and facilitating appropriately timed examinations and treatments.

## Supporting information

S1 File (XLSX)
